# Longitudinal analysis of hepatic transcriptome and serum metabolome demonstrates altered lipid metabolism following the onset of hyperglycemia in spontaneously diabetic biobreeding rats

**DOI:** 10.1371/journal.pone.0171372

**Published:** 2017-02-13

**Authors:** Simon E. Regnell, Martin J. Hessner, Shuang Jia, Lina Åkesson, Hans Stenlund, Thomas Moritz, Daria La Torre, Åke Lernmark

**Affiliations:** 1 Diabetes and Celiac Disease Unit, Department of Clinical Sciences, Lund University/Clinical Research Centre and Skåne University Hospital, Malmö, Sweden; 2 Max McGee National Research Center for Juvenile Diabetes, Children's Research Institute of Children's Hospital of Wisconsin, Medical College of Wisconsin, Milwaukee, Wisconsin, United States of America; 3 Swedish Metabolomics Centre, Swedish University of Agricultural Sciences, Umeå, Sweden; East Tennessee State University, UNITED STATES

## Abstract

Type 1 diabetes is associated with abberations of fat metabolism before and after the clinical onset of disease. It has been hypothesized that the absence of the effect of insulin in the liver contributes to reduced hepatic fat synthesis. We measured hepatic gene expression and serum metabolites before and after the onset of hyperglycemia in a BioBreeding rat model of type 1 diabetes. Functional pathway annotation identified that lipid metabolism was differentially expressed in hyperglycemic rats and that these pathways significantly overlapped with genes regulated by insulin. 17 serum metabolites significantly changed in concentration. All but 2 of the identified metabolites had previously been reported in type 1 diabetes, and carbohydrates were overall the most upregulated class of metabolites. We conclude that lack of insulin in the liver contributes to the changes in fat metabolism observed in type 1 diabetes. Further studies are needed to understand the clinical consequences of a lack of insulin in the liver in patients with type 1 diabetes.

## Introduction

Type 1 diabetes is characterized by the autoimmune destruction of the pancreatic beta cells, causing a deficiency of insulin. The clinical onset of diabetes is preceded by a period with circulating islet autoantibodies that can last for years [1]. In addition, long before the appearance of autoantibodies, differences in the serum levels of certain lipids can be measured in persons who go on to develop type 1 diabetes compared to controls. These differences appear to exist already *in utero*, as umbilical cord blood levels of phosphatidylcholines and phosphatidylethanolamines were significantly decreased in children diagnosed with type 1 diabetes before 4 years of age [2]. The serum metabolite profiles of children who progressed to type 1 diabetes and controls who remained permanently autoantibody negative and nondiabetic have been studied longitudinally [3]. Persons who developed diabetes had reduced serum levels of succinic acid and phosphatidylcholine at birth, reduced levels of triglycerides and antioxidant ether phospholipids throughout the follow up, and increased levels of proinflammatory lysophosphatidylcholines several months before seroconversion to autoantibody positivity. The lipid profiles in the progressors during the last visits before diagnosis of type 1 diabetes revealed no differences as compared to the profiles of matched nonprogressors apart from differences that were already apparent from previous samples. However, due to the long periods between samples, it is yet unclear whether any changes in serum lipids take place close to the clinical onset of type 1 diabetes.

Fat metabolism is also perturbed after the diagnosis of type 1 diabetes. Patients have less fat in the liver [4] and increased fasting lipid oxidation [5] compared to controls. Similarly, BioBreeding diabetes-prone (BBDP) rats develop a reduced respiratory quotient compared to non-diabetic rats before the onset of hyperglycemia, consistent with an increased use of fatty acids relative to carbohydrates as an energy substrate [6].

The type 1 diabetes-like non-obese diabetic (NOD) mouse, which develops peri-insular mononuclear cell infiltration weeks prior to diabetes, showed reduced circulating polyunsaturated fatty acids and lipid signaling mediators; hypertriglyceridemia; and decreased lysophosphatidylcholines and phosphatidylcholines [7, 8]. In NOD mice developing diabetes, metabolomics analyses showed increased plasma glucose and reduced 1,5-anhydroglucitol; increased allantoin, gluconic acid and nitric oxide-derived saccharic acid markers of oxidative stress; changes in certain amino acids; and reduced unsaturated fatty acids, including arachidonic acid [9]. The prolonged prodrome of diabetes onset in humans and the NOD mouse and alterations in plasma metabolites may reflect different stages in disease development. As humans with multiple islet autoantibodies rarely have signs of insulitis [10, 11], it would be critical to test whether acute loss of insulin as opposed to a slowly progressing loss would result in a different liver response.

We hypothesized that a lack of insulin reaching the liver contributes to the metabolic shift towards lipid oxidation observed in humans with type 1 diabetes and rodent models of the disease. To test our hypothesis, we measured changes in the hepatic gene expression and serum metabolome of congenic BBDR.lyp rats that spontaneously develop diabetes within 1–2 days without sign of prior insulitis [12–14]. We found that hepatic gene expression relating to lipid metabolism was significantly changed following disease onset and that there was a significant overlap with genes regulated by insulin.

## Materials and methods

### Animals

The BioBreeding Diabetes Resistant (BBDR) rat made homozygous for the lymphopenia (*lyp*) mutation after introgression of the insulin-dependent diabetes mellitus (*iddm*) *1* region from the BBDP rat was obtained by sister-brother breeding of heterozygous BBDR.^*lyp/+*^ rats [15]. Inheritance of the *lyp* gene is Mendelian, and all BBDR.^*lyp/lyp*^ rats develop diabetes. BBDR.^*lyp/lyp*^ rats develop insulitis and rapidly lose beta cells 1–2 days before the sudden onset of hypoinsulinemia, hyperglycemia, weight loss, and ketonuria at 46 to 81 days after birth. BBDR.^*lyp*/+^ and BBDR.^+/+^ rats are diabetes-resistant and are phenotypically and biochemically indistinguishable from each other [16].

The rats (BBDR.^*lyp/lyp*^ n = 17; BBDR.^*lyp*/+^ n = 12; BBDR.^+/+^ n = 11) were housed in specific pathogen-free conditions at 21°C with 12 hour cycles of light and darkness and free access to food (R3 chow, Lactamin, Kimstad, Sweden) and water. Rats were housed at 21–23°C at the Clinical Research Centre facility in Malmö, Sweden, with 2 to 6 rats per cage. Experiments were approved by the Animal Ethical Committee in Lund, Sweden (permit number M152-08) and followed the Guide for the Care and Use of Laboratory Animals of the National Institutes of Health [17]. Sample collection was performed under isoflurane anesthesia, and all efforts were made to minimize suffering. Capillary glucose measurements were performed daily at between 8 AM and 10 AM. Hyperglycemia was defined as capillary glucose ≥ 11.1 mmol/L.

The rats were genotyped with microsatellites using DNA from ear punches to identify rats predestined to develop diabetes (BBDR.^*lyp/lyp*^) or to remain diabetes resistant (BBDR.^*lyp*/+^ and BBDR.^+/+^) [14, 15]. Venous samples (100–500 μl) were drawn from the tail vein every fifth day from 40 days and every third day from 50 days between 10 AM and 1 PM. Venous sampling continued until one sample had been obtained during hyperglycemia in DP rats and was performed in parallel in the DR littermates, providing about 7 venous samples per rat (Table 1). For a smaller number of rats, hepatic tissue was excised at approximately 40, 50, and 60 days after birth, providing two measurements before becoming hyperglycemic and one within a day of DP rats becoming hyperglycemic. Hepatic tissue was immediately frozen in liquid nitrogen. Serum and hepatic samples were stored at -80°C. In accordance with the ethical permit, the BBDR.^*lyp/lyp*^ rats were sacrificed by CO_2_ inhalation before the onset of ketoacidosis, on the first or second day of hyperglycemia, after a final liver and blood sample had been obtained.

**Table 1:** Schematic overview of venous and hepatic sample acquisition. Samples obtained at day 60 represent the average onset of hyperglycemia—the final samples in BBDR.^*lyp/lyp*^ rats were always obtained after the rats had become hyperglycemic. BBDR.^*lyp/ly*^ n = 17, BBDR.^*lyp/+*^ n = 12, BBDR.^*+/+*^ n = 11.

**Venous sample**	X	X	X	X	X	X	X
**Hepatic sample**	X		X				X
**Day**	40	45	50	53	56	59	60

### Hepatic gene expression and signaling pathway analysis

Total liver RNA was extracted with Trizol (Invitrogen, Carlsbad, USA). RNA (~100 ng) was amplified and labeled (Affymetrix two-cycle cDNA synthesis kit, Affymetrix, Santa Clara, USA) and hybridized to the Affymetrix RG230 2.0 array per the manufacturers’ protocol. Array images were quantified with Affymetrix Expression Console Software and normalized and analyzed with Partek Genomic Suite (Partek Inc, St. Louis, USA) to determine signal log ratios. The average gene expression from the two hepatic samples before hyperglycemia was compared with the expression from a single sample obtained during hyperglycemia. The statistical significance of differential gene expression was determined with a Student’s t test and false discovery rates. Gene expression differences were evaluated by principal component analyses as well as non-parametric rank product tests to assess the rate of type I errors in multiple testing (www.bioconductor.org) [18, 19]. Findings with a rank product false discovery rate < 20% were considered of interest. Ontological analyses were conducted with the Database for Annotation, Visualization, and Integrated Discovery version 6.7 (DAVID) [20] and the Ingenuity Pathway Analysis (IPA) package (Ingenuity Systems, Redwood City, USA). Hierarchical clustering was conducted with Genesis [21]. Data files are available at The National Center for Biotechnology Information Gene Expression Omnibus (accession number: GSE84886).

### Gas chromatography–mass spectrometry analysis of blood samples

Metabolites were extracted from serum samples and analyzed as described previously [22]. Briefly, 70 μl of serum was diluted with 630 μl of methanol diluted volume 9:1 in water including internal standards. After centrifugation at 19600 g for 10 minutes at 4°C, 200 μl of the supernatant was transferred to a gas chromatography–mass spectrometry vial and evaporated until dry.

Derivatized sample (1 μl) was transferred by an Agilent 7683 Series Autosampler (Agilent, Atlanta, USA) into an Agilent 6980 gas chromatography device that had a 10 m x 0.18 mm ID, fused silica capillary column chemically bonded 0.18 μm DB5 mass spectrometry stationary phase (J&W Scientific, Folsom, USA). The injector temperature was 270°C. Helium was used as carrier gas with a flow rate of 1 ml/minute. Column temperature was initially 70°C. After 2 minutes, the temperature was increased by 40°C/minute until it reached 320°C, which was maintained for 2 minutes. The column effluent was entered into the ion source of a Pegasus III TOFMS (Leco Corp., St. Joseph, USA). The transfer line temperature was 250°C and ion source temperature was 200°C. Ions were generated by a 70 eV electron beam at a current of 2.0 mA. Masses were acquired from mass/charge 50 to 800 at a rate of 30 spectra/second.

Unprocessed mass spectrometry files were exported from ChromaTOF software (Leco Corp., St Joseph, USA) in NetCDF format to MATLAB 8.5 (Mathworks, Natick, USA). Custom scripts were used for data pre-treatment. 90 compounds were detected in each of the blood samples. Metabolites were identified by comparing retention indices and mass spectra with in-house mass spectrum libraries [23]. Principal component analysis was used to get an overview of the data and orthogonal projections to latent structures for discriminant analysis was used for class prediction, as described previously [22]. Multivariate modeling was performed in SIMCA Version 14.0.0.1359 (Umetrics, Umeå, Sweden).

## Results

### Rat characteristics

**Fig 1:**
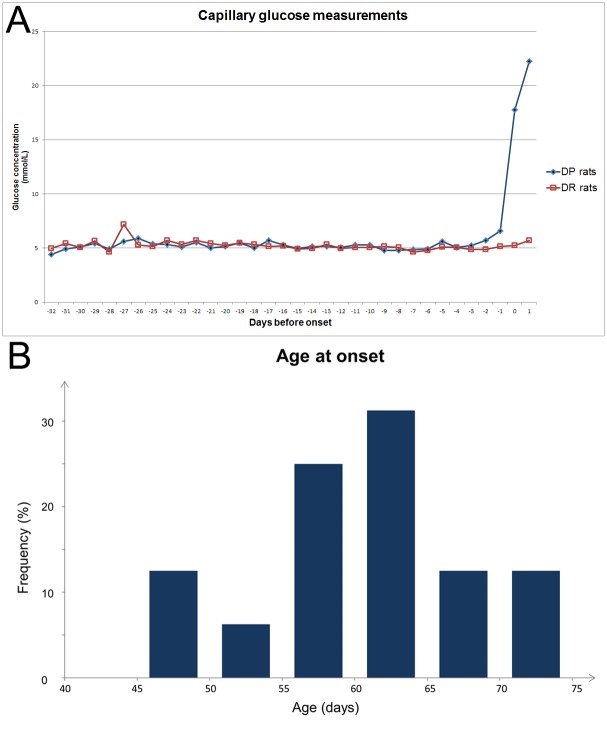
(A) Comparison of capillary glucose levels in DP (BBDR.^*lyp/lyp*^) rats before and after the onset of hyperglycemia and DR (BBDR.^*lyp/+*^ and BBDR.^+/+^) rats at comparable ages. (B) Histogram showing the distribution of age at hyperglycemia onset for BBDR.^*lyp/lyp*^ rats.

A significant difference in average blood glucose between DP (*lyp/lyp*) and DR (*lyp/+* and *+/+*) animals was detected 2 days before the onset of hyperglycemia (5.7 versus 4.9 mmol/L; p < 0.001). In DP rats, mean capillary glucose was 17.8 mmol/L (standard deviation 4.3 mmol/L) on the first day of hyperglycemia and 22.3 mmol/L (standard deviation 5.3 mmol/L) on the second day of hyperglycemia (Fig 1A). Average age at hyperglycemia onset was 60 days (standard deviation 7.3 days). The distribution of the age of hyperglycemia onset is illustrated as a histogram in Fig 1B. The weight of DP and DR rats was indistinguishable until shortly before diabetes onset, when the DP rats started losing weight. For males, average weight at hyperglycemia onset was 275 g (standard deviation 16 g) for DP rats and 295 g (standard deviation 9.9 g) for DR rats at the corresponding age (p = 0.05). For females, average weight at hyperglycemia onset was 174 g (standard deviation 22 g) for DP rats and 198 g (standard deviation 12.1 g) for DR rats at the corresponding age (p = 0.03).

### Functional annotation of hepatic gene expression and canonical pathway analysis

**Fig 2:**
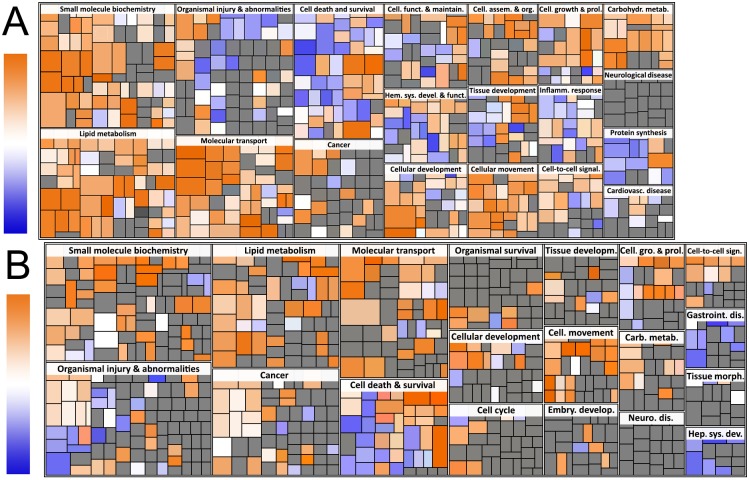
Functional classification of altered metabolic pathways in the hepatic transcriptome. The size of each rectangle is proportional to–log(p value) of the pathway that the rectangle represents. Red signifies upregulation, while blue signifies downregulation in hyperglycemic animals. (A) BBDR.^*lyp/lyp*^ post-hyperglycemia versus BBDR.^+/+^ rats of the same age. (B) BBDR.^*lyp/lyp*^ pre-hyperglycemia versus BBDR.^*lyp/lyp*^ post-hyperglycemia.

We limited the gene expression analysis to comparing non-hyperglycemic (samples from 40 and 50 days of age in DP rats, and 40, 50, and 60 days of age in DR rats) and hyperglycemic rats (average age 60 days in DP rats). By separately comparing BBDR.^*lyp/lyp*^ pre-hyperglycemia with BBDR.^*lyp/lyp*^ post-hyperglycemia onset and BBDR.^*lyp/lyp*^ post-hyperglycemia onset with BBDR.^*lyp/+*^ and BBDR.^+/+^ of the same age (average 60 days), we reduced the risk of type I error. The data in Fig 2 display the most significantly altered genetic pathways according to functional classification. In addition to pathways related to inflammation, lipid metabolism was identified as one of the most significantly altered pathways.

To further characterize changes in hepatic gene expression related to lipid metabolism, functional annotations subsumed under *lipid metabolism* were identified in IPA. Results with consistent activation scores in both BBDR.^*lyp/lyp*^ pre-hyperglycemia versus during hyperglycemia and BBDR.^*lyp/lyp*^ during hyperglycemia versus BBDR.^*lyp/+*^ and BBDR.^*+/+*^ at the same age are displayed in Table 2 (p value for both, independent analyses < 0.006).

**Table 2:** Functional annotation of pathways consistently upregulated within *lipid metabolism* in both BBDR.^*lyp/lyp*^ pre-hyperglycemia versus post-hyperglycemia and BBDR.^*lyp/lyp*^ post-hyperglycemia versus BBDR.^*lyp/+*^ and BBDR.^*+/+*^ at the same age, with their associated genes.

Functional annotation	Activation score	Genes
Hepatic steatosis	Negative	*Acaca*, *Adk*, *Agtr1*, *Bhlhe40*, *Cav1*, *Ccnd1*, *Cd14*, *Cd36*, *Cdkn1a*, *Cpt1a*, *Dusp1*, *Ehhadh*, *Elovl5*, *G6pc*, *Ghr*, *Gpd1*, *Ifnar1*, *Lrp6*, *Mut*, *Por*
Transport of lipid	Positive	*Abcb1b*, *Abcb4*, *Abcg8*, *App*, *Canx*, *Cav1*, *Cd14*, *Cd36*, *Cpt1a*, *Crot*, *Cyp7a1*, *Gdf15*, *Ghr*, *Osbpl1a*, *Reln*, *Scarb1*, *Slc9a3r1*, *Slco1a4*, *Slco2a1*
Transport of steroid	Positive	*Abcb1b*, *Akr1c4*, *Apoe*, *App*, *Canx*, *Cav1*, *Cd36*, *Msn*, *Osbp*, *Osbpl1a*, *Reln*, *Rhoa*, *S100a8*, *S100a9*, *Scarb1*, *Scd*, *Slc9a3r1*, *Slco1a4*, *Slco1b3*, *Srebf1*
Oxidation of lipid	Positive	*Acaca*, *Acacb*, *Acadsb*, *Acox3*, *Adh4*, *Akt2*, *App*, *Cd36*, *Cpt1a*, *Crot*, *Ehhadh*, *Gcdh*, *Ide*, *Lrpprc*, *Por*, *Scarb1*, *Slco2a1*
Transport of sterol	Positive	*Abcb1b*, *Apoe*, *App*, *Canx*, *Cav1*, *Cd36*, *Msn*, *Osbp*, *Osbpl1a*, *Reln*, *Rhoa*, *S100a8*, *S100a9*, *Scarb1*, *Scd*, *Slc9a3r1*, *Srebf1*
Oxidation of fatty acid	Positive	*Acaca*, *Acacb*, *Acadsb*, *Acox3*, *Adh4*, *Akt2*, *Cd36*, *Cpt1a*, *Crot*, *Ehhadh*, *Gcdh*, *Ide*, *Lrpprc*, *Por*, *Slco2a1*
Export of lipid	Positive	*Apoe*, *App*, *Canx*, *Cav1*, *Cd36*, *Crot*, *Ghr*, *Msn*, *Reln*, *Rhoa*, *S100a8*, *S100a9*, *Scarb1*, *Scd*, *Srebf1*
Efflux of cholesterol	Positive	*Abcb4*, *Abcg8*, *App*, *Canx*, *Cav1*, *Cd36*, *Cyp7a1*, *Gdf15*, *Reln*, *Scarb1*
Efflux of lipid	Positive	*Abcb4*, *Abcg8*, *App*, *Canx*, *Cav1*, *Cd14*, *Cd36*, *Cyp7a1*, *Gdf15*, *Ghr*, *Reln*, *Scarb1*
Synthesis of steroid	Positive	*Abcg8*, *Agtr1*, *App*, *Atp1a1*, *Bre*, *Cav1*, *Cyp39a1*, *Cyp7a1*, *G6pc*, *Gdf15*, *Igfbp2*, *Il1a*, *Inhba*, *Nr1d1*, *Por*, *Prlr*, *Scarb1*, *Sec14l2*, *Slc9a3r1*, *Wnt4*
Fatty acid metabolism	Positive	*Abat*, *Abcb1b*, *Abcb4*, *Abcg8*, *Acaca*, *Acacb*, *Acadsb*, *Acot12*, *Acot2*, *App*, *Asah1*, *Canx*, *Cav1*, *Cd14*, *Cd36*, *Cpt1a*, *Crot*, *Cxcl12*, *Cyp7a1*, *Dab1*, *Ehhadh*, *Elovl5*, *Elovl6*, *Far1*, *Gcdh*, *Gdf15*, *Ghr*, *Gnai3*, *Hnf4a*, *Il1a*, *Lyn*, *Mapk9*, *Mgll*, *Osbpl1a*, *Por*, *Reln*, *Scarb1*, *Sgpl1*, *Slc9a3r1*, *Slco1a4*, *Slco2a1*
Concentration of cholesterol	Positive	*Abcb4*, *Abcg8*, *Acacb*, *App*, *Atp1a1*, *Cav1*, *Cd36*, *Cdkn1a*, *Cyp7a1*, *Dicer1*, *Dusp1*, *Entpd5*, *G6pc*, *Ghr*, *Gulo*, *Hnf4a*, *Mgll*, *Nampt*, *Por*, *Rab7a*, *Scarb1*, *Sec14l2*, *Sigmar1*
Synthesis of lipid	Positive	*Abat*, *Abcb1b*, *Abcg8*, *Acaca*, *Acacb*, *Adh4*, *Agtr1*, *App*, *Arf1*, *Asah1*, *Atp1a1*, *Bre*, *Cav1*, *Cd14*, *Cd36*, *Cxcl12*, *Cyp26a1*, *Cyp39a1*, *Cyp7a1*, *Dab1*, *Elovl5*, *Elovl6*, *Far1*, *G6pc*, *Gcdh*, *Gdf15*, *Ghr*, *Gnai3*, *Hnf4a*, *Igfbp2*, *Il1a*, *Inhba*, *Lyn*, *Mapk9*, *Nr1d1*, *Pi4k2a*, *Por*, *Prlr*, *Scarb1*, *Sec14l2*, *Sgpl1*, *Slc6a6*, *Slc9a3r1*, *Wnt4*
Concentration of sterol	Positive	*Abcb4*, *Abcg8*, *Acacb*, *App*, *Atp1a1*, *Cav1*, *Cd36*, *Cdkn1a*, *Cyp7a1*, *Dicer1*, *Dusp1*, *Entpd5*, *G6pc*, *Ghr*, *Gulo*, *Hnf4a*, *Mgll*, *Nampt*, *Por*, *Rab7a*, *Scarb1*, *Sec14l2*, *Sgpl1*, *Sigmar1*, *Slc9a3r1*
Secretion of lipid	Positive	*Abat*, *Abcb4*, *Abcg8*, *Asah1*, *Bhlhe40*, *Cd36*, *Ctnnb1*, *Cyp7a1*, *Il1a*, *Inhba*, *Mapk9*, *Mgll*, *Scarb1*

Cellular signaling pathways potentially regulating hepatic gene expression were identified through IPA canonical pathway analysis. The ten pathways most significantly altered pathways in each comparison (including only those that were consistently and significantly differentially expressed in both comparisons) are displayed in Table 3.

**Table 3:** Ten most significantly differentially expressed canonical pathways in BBDR.^*lyp/lyp*^ post-hyperglycemia versus BBDR.^*lyp/+*^ and BBDR.^*+/+*^ and BBDR.^*lyp/lyp*^ post-hyperglycemia versus BBDR.^*lyp/lyp*^ pre-hyperglycemia.

*+/+* versus *lyp/lyp* pathways	-log(p-value)	z-score	*lyp/lyp* pre- versus post-hyperglycemia	-log(p value)	z-score
Chemokine receptor type 4 Signaling	3.35	3.207	AMP-activated protein kinase signaling	3.95	-0.632
Hypoxia signaling in the cardiovascular system	3.34	Activity pattern unavailable	Remodeling of epithelial adherens junctions	3.63	1.89
Pregnane X receptor/ retinoid X receptor activation	3.23	Activity pattern unavailable	Phosphoinositide 3-kinase/Akt signaling	3.47	1.265
Epithelial adherens junction signaling	3.17	Activity pattern unavailable	Wnt/β-catenin signaling	2.96	1
Fcγ receptor-mediated phagocytosis in macrophages and monocytes	3.12	1.508	Epithelial adherens junction signaling	2.90	Activity pattern unavailable
Production of nitric oxide and reactive oxygen species in macrophages	2.90	1.807	p70S6K signaling	2.82	1
AMP-activated protein kinase signaling	2.88	-0.577	Pyridoxal 5'-phosphate salvage pathway	2.75	Activity pattern unavailable
Signaling by rho family GTPases	2.87	3.873	Pregnane X receptor/ retinoid X receptor activation	2.13	Activity pattern unavailable
Phosphoinositide 3-kinase/Akt signaling	2.80	2.309	Rho GDP-dissociation inhibitor signaling	1.90	-1.89
Inhibition of angiogenesis by thrombospondin 1	2.75	2.449	Production of nitric oxide and reactive oxygen species in macrophages	1.88	0.632

### Insulin and glucose regulator effects

**Fig 3:**
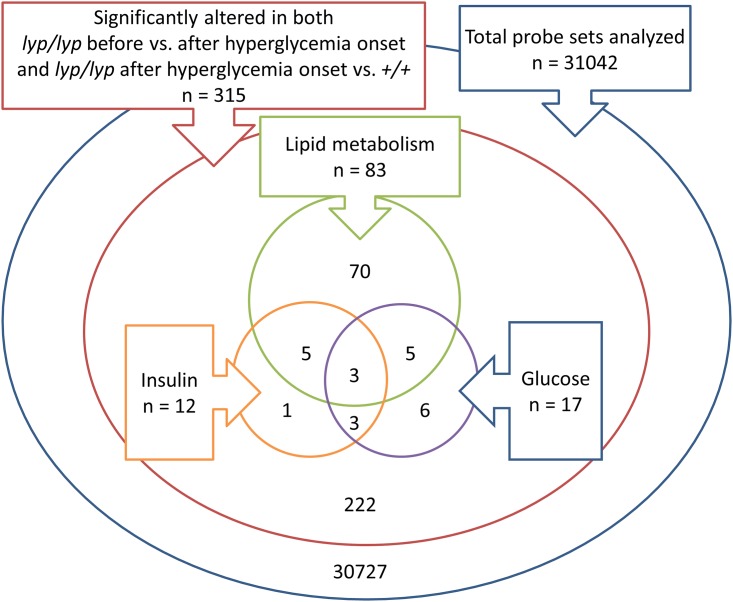
Of all the probe sets analyzed (n = 31,042), 315 were altered in a consistent direction when comparing BBDR.^*lyp/lyp*^ before hyperglycemia with BBDR.^*lyp/lyp*^ after hyperglycemia and BBDR.^*lyp/lyp*^ after hyperglycemia with BBDR.^*lyp/+*^ and BBDR.^+/+^ at the same age. Of these 315 genes, 83 were identified in pathways related to lipid metabolism. 12 of the 315 the genes were regulated by insulin, of which 8 overlapped with the 83 genes related to lipid metabolism. 17 of the 315 genes were regulated by glucose, of which 8 overlapped with the 83 genes related to lipid metabolism.

We had hypothesized that altered hepatic lipid metabolism is caused by the absence of insulin, and insulin was identified by IPA in both comparisons as a differentially expressed upstream regulator of the observed patterns of hepatic gene expression. Genes regulated by insulin were therefore identified in IPA. The same was done for genes regulated by glucose, which is the obvious metabolic perturbation in the BBDR.^*lyp/lyp*^ rats. Genes that are regulated by insulin and/or glucose and are significantly altered in BBDR.^*lyp/lyp*^ before and after the onset of hyperglycemia as well as in BBDR.^*lyp/lyp*^ post-hyperglycemia compared to BBDR.^*lyp/+*^ and BBDR.^*+/+*^ at the corresponding age are displayed in Table 4. Genes that overlap with the canonical pathways listed in Table 2 are noted. 8 of the 12 genes regulated by insulin were also independently identified in the functional notation analysis (p value for the overlap = 0.0033, calculated as the hypergeometric probability). 8 of 17 genes regulated by glucose were identified in the functional notation analysis (p = 0.048). The relationship between the total genes analyzed, the significantly altered genes, the genes related to lipid metabolism, the genes regulated by insulin, and the genes regulated by glucose are visualized in Fig 3.

**Table 4:** Genes consistently altered in both in BBDR.^*lyp/lyp*^ before versus after the onset of hyperglycemia and in BBDR.^*lyp/lyp*^ post-hyperglycemia versus to BBDR.^*lyp/+*^ and BBDR.^*+/+*^ that are regulated by insulin and/or glucose.

Gene	Insulin p = 1.27E-4	D-glucose p = 3.17E-3	Fold change BBDR.^*lyp/lyp*^ before and after hyperglycemia onset	Fold change BBDR.^*lyp/lyp*^ versus BBDR.^*+/+*^	Found in altered functional annotation?
*Cd36*	Yes	Yes	1.7	1.9	Yes
*Cdkn1a*	Yes	Yes	-1.7	-1.7	Yes
*Actb*	Yes	Yes	1.7	2.0	No
*G6pc*	Yes	Yes	2.0	2.0	Yes
*Agtr1a*	Yes	Yes	1.8	1.8	No
*Uqcrc2*	Yes	Yes	2.0	2.5	No
*App*	Yes	No	1.7	2.3	Yes
*Ide*	Yes	No	1.6	2.1	Yes
*Acot12*	Yes	No	1.6	1.7	Yes
*Gclc*	Yes	No	1.6	2.0	No
*Scarb1*	Yes	No	2.1	1.7	Yes
*Dusp1*	Yes	No	2.6	4.0	Yes
*Canx*	No	Yes	1.9	2.7	Yes
*Atp6ap2*	No	Yes	1.7	1.7	No
*Cap1*	No	Yes	1.6	1.9	No
*Gcdh*	No	Yes	1.6	1.9	Yes
*Pdcd4*	No	Yes	1.7	1.7	No
*Txnrd1*	No	Yes	1.7	2.3	No
*Abcb1b*	No	Yes	1.8	1.7	Yes
*Cav1*	No	Yes	1.7	1.7	Yes
*Gpd1*	No	Yes	1.7	2.4	Yes
*Sec61a1*	No	Yes	1.9	2.0	No
*Glul*	No	Yes	2.0	1.7	No

### Metabolites

**Fig 4:**
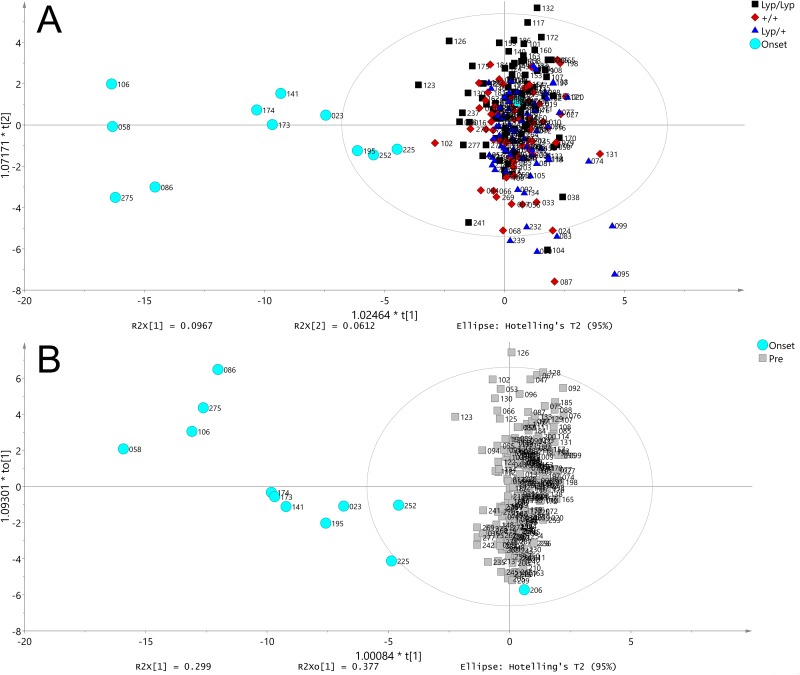
Orthogonal projections to latent structures for discriminant analysis plot of measured metabolites. The numbers next to each symbol represents the sample identifier. (A) Comparison of metabolites from BBDR.^*lyp/lyp*^ (black) pre-hyperglycemia, BBDR.^*lyp*/+^ (dark blue), BBDR.^+/+^ (red) rats, and BBDR.^*lyp/lyp*^ post-hyperglycemia (light blue). 236 samples and 78 annotated metabolites are included in the model with 3 Y variables. (B) Model with 2 classes and 1 Y variable. Through model optimization, 19 metabolites that distinguished BBDR.^*lyp/lyp*^ after hyperglycemia onset (light blue) from others (gray) were identified according to predictive loadings and 95% confidence interval. Of these, 1 metabolite (maltose) was removed as a duplicate and 1 metabolite (citrulline) was removed as a false positive finding due to its degradation products being identical to those of other amino acids.

None of the measured metabolites could be used to distinguish between the different genotypes before the onset of hyperglycemia, (Fig 4A). Comparing the metabolite profile of BBDR.^*lyp/lyp*^ rats after the onset of hyperglycemia with other samples identified 17 metabolites that significantly differed in BBDR.^*lyp/lyp*^ rats post-hyperglycemia compared to other BBDR.^*lyp/lyp*^ pre-hyperglycemia, BBDR^lyp/+^, and BBDR^+/+^, as demonstrated in Fig 4B and Table 5. Values for fold change were normalized in relation to sucrose, which was used as an internal standard. Glucose was not included, as the region was maximally saturated. Data for each metabolite at each sample time point for each rat is included in S1 File.

**Table 5:** Metabolites that distinguish BBDR.^*lyp/lyp*^ rats post-hyperglycemia from other genotypes and BBDR.^*lyp/lyp*^ pre-hyperglycemia. Metabolites were analysed as MeOxTMSi derivatives.

Metabolite	Class	Average fold change (normalized)	Uncorrected p value	Previously published alterations in type 1 diabetes
Unidentified	Carbohydrate	1.9 (1.8)	0.000055	
Unidentified	Carbohydrate	2.2 (2.0)	0.001298	
Unidentified	Carbohydrate	1.3 (1.2)	0.000159	
Unspecified hexose	Carbohydrate	0.5 (0.5)	0.000029	
Unidentified	Carbohydrate	1.4 (1.4)	0.000350	
Laminaribiose	Carbohydrate	1.1 (1.1)	0.001598	No
Maltose	Carbohydrate	1.6 (1.5)	0.002172	Yes [7, 9, 24]
Unspecified disaccharide	Carbohydrate	1.2 (2.2)	0.004414	
Linoleic acid	Fatty acid	0.6 (0.5)	0.027734	Yes [25]
Oleic acid	Fatty acid	0.7 (0.7)	0.011016	Yes [7, 24, 25]
Citric acid	Carboxylic acid	-0.2 (-0.2)	0.009388	Yes [24–28]
Scyllo-inositol	Carbohydrate	-0.3 (-0.3)	0.000768	No
Ornithine	Amino acid	-0.4 (-0.4)	0.00054	Yes [24, 27, 29]
Hippuric acid	Carboxylic acid	-0.5 (-0.5)	0.000004	Yes [26, 28, 30]
Tyrosine	Amino acid	-0.6 (-0.4)	0.000121	Yes [7, 24, 27, 30, 31]
Lysine	Amino acid	-0.6 (-0.6)	4.064 E-8	Yes [9, 24, 25, 27]
1,5-anhydro-D-glucitol	Carbohydrate	-0.9 (-0.8)	8.4704 E-11	Yes [7, 9, 32]

## Discussion

Studies in patients with type 1 diabetes have shown lower levels of liver fat compared to controls in both children and adults [4, 5]. Obese patients with type 1 diabetes are resistant against fatty liver disease [33]. Patients with type 1 diabetes have abnormal lipoprotein patterns, even if they have optimal glycemic control [34]. Based on these findings, which are unrelated to diabetes duration, we expected hepatic lipid metabolism to change during the transition from normoglycemia to hyperglycemia in BBDR.^*lyp/lyp*^ rats. The results of the functional annotation analysis of the hepatic transcriptome support our hypothesis that hepatic fat anabolism is impaired during the onset of type 1 diabetes. When comparing both BBDR.^*lyp/lyp*^ rats before hyperglycemia versus during hyperglycemia and BBDR.^*lyp/lyp*^ rats during hyperglycemia versus BBDR.^*lyp/+*^ and BBDR.^+/+^ rats, lipid metabolism was identified as the most significantly altered physiological function besides inflammation in terms of the magnitude of transcript changes and their statistical significance. The functional annotation *hepatic steatosis* was reduced, whereas other lipid processes were enhanced.

It has been unclear whether aberrations of fat metabolism in type 1 diabetes are caused by the absence of insulin in the liver, by decreased fatty acid substrate availability in the blood secondary to peripheral hyperinsulinemia due to subcutaneous insulin injections, or by a combination of both [35]. Our upstream regulation and canonical pathway analysis showed a significant overlap between genes regulated by insulin and genes identified by functional annotation as related to fat metabolism, suggesting that lack of insulin action in the liver affects fat metabolism at least partially independently of peripheral effects on fat metabolism at the onset of type 1 diabetes. Further studies would be needed to determine the mechanisms underlying peripheral alterations of fat metabolism in the natural history of type 1 diabetes and whether there are any interactions with the hepatic abberations that we observed. The overlap we observed between the differentially expressed genes and genes regulated by glucose suggests that increased blood glucose levels per se might also influence hepatic lipid metabolism.

A number of pathways known to be affected by insulin, such as phosphoinositide 3-kinase/Akt and p70S6K, as well as regulators of hepatic lipogenesis, such as AMP-activated protein kinase and pregnane X receptor signalling, were identified [36]. Furthermore, some insulin-regulated genes known to influence hepatic lipogenesis (such as acetyl-CoA carboxylase) were differentially expressed according to predefined thresholds for false discovery rate in one, but not both, comparisons. A limitation of our methodology of considering only genes differentially expressed in two comparisons is that this unusually strict methodology may cause false some negative results. It was also recently found that the phosphoinositide 3-kinase pathway in T cells from BBDP rats may be constitutively activated; it is therefore conceivable that this pathway is abberently expressed also in the liver in the rat model used, which would have to be taken into consideration when interpreting any findings related to the pathway [37]. Also, microarray and metabolite analyses can be susceptible to batch effects, which can also impact the generalizability of the findings. Further studies employing methods complementary to microarray analysis, such as polymerase chain reaction techniques, could use a more targeted approach to specifically test the genes that we identified as differentially expressed, thereby ensuring the generalizability of our findings.

In addition to longitudinally analyzing hepatic gene expression, we studied how serum metabolites changed during the corresponding time points. A previous analysis of the serum metabolome of the two types of rats showed that a metabolic signature appears in the BBDR.^lyp/lyp^ rat before hyperglycemia, including alterations in fatty acids, phospholipids, and amino acids [22]. In contrast, we could not detect any differences in metabolites between BBDR.^+/+^ rats and BBDR.^lyp/lyp^ before the onset of hyperglycemia. The nature of metabolomics experiments typically necessitates repeated studies to determine whether results represent true positive findings. Numerous environmental factors, as well as differences between the individual rats, may have caused the varying results. Further studies with other populations will have to be performed before any metabolite alterations before or after the onset of type 1 diabetes can be confirmed.

In our metabolite analysis, carbohydrates were the most clearly increased class of metabolites, consistent with an overall increase in plasma carbohydrates found in diabetic NOD mice [9]. Most of the metabolites that we found were altered during hyperglycemia have previously been identified in type 1 diabetes or animal models thereof, including in patients with a short disease duration and patients temporarily deprived of exogenous insulin, which increases the validity of our results. We found reduced levels of 1,5-anhydro-D-glucitol, which is a well-established marker of glycemic excursions [38]. Higher levels of citric and hippuric acid have been reported in the urine of children with type 1 diabetes, possibly due to an increased glomerular filtration rate in the early stages of the disease [26].

Changes in plasma amino acid levels that correlate to increasing hyperglycemia have been described in insulin-deficient mice [39], and type 1 diabetes patients have been shown to have increased excretion of lysine in their urine [25]. Ornithine decarboxylase, which catalyzes the decarboxylation of ornithine, is increased in rodents with streptozotocin-induced diabetes [40], and increased activity of ornithine decarboxylase has been linked to a transient increase of glomerular filtration rate in early diabetes [41]. Increased activity of ornithine decarboxylase thus provides a possible explanation for our finding reduced levels of ornithine.

There are possible biological links between the changes in gene expression and those in metabolites that we uncovered. For instance, several genes in the oleate synthesis pathway were affected, and serum oleate concentrations were increased by about 50%. Overall, though, we are cautious about theorizing about specific chemical compounds, as our purpose with this experiment was to examine broader changes in metabolism. The patterns of hepatic gene expression that we found suggest that type 1 diabetes has complex and dynamic effects on fat metabolism, which would not be reflected in the metabolite profile at a single timepoint. Studies of the metabolite flux into, out of, and within the liver could further clarify the role of the liver in the metabolic changes that take place at the beginning of type 1 diabetes.

## Conclusions

Our study suggests that the shift towards fat oxidation occurs early in the course of type 1 diabetes and is affected by the loss of insulin action in the liver. Further studies are needed to clarify the clinical consequences of a lack of insulin in the liver for patients with type 1 diabetes.

## Supporting information

S1 File Metabolite data from gas chromatography-mass spectrometry analysis. (XLSX)
